# In vitro anti-*Toxoplasma gondii* activity of *Ganoderma lucidum* extracts

**DOI:** 10.1186/s13104-023-06355-6

**Published:** 2023-05-18

**Authors:** Mohammad ahmadi, Mahboobeh Salimi, Mehrzad Saraei, Niloofar Salavati Nezhad, Amir Javadi, Faezeh Mohammadi, Peyman Heydarian, Ehsan Ali, Elham Hajialilo

**Affiliations:** 1 Student Research Committee, Qazvin University of Medical Sciences, Qazvin, Iran; 2 Department of medical parasitology and Mycology, Tehran University of Medical Sciences, Tehran, Iran; 3 Medical Microbiology Research Center, Qazvin University of Medical Sciences, Qazvin, Iran; 4 Department of Parasitology and Mycology, Qazvin University of Medical Sciences, Qazvin, Iran; 5 Department of medicine Biotechnology, Faculty of Allied Medicine, Qazvin University of Medical Sciences, Qazvin, Iran; 6 Department of Community Medicine, School of Medicine, Qazvin University of Medical Sciences, Qazvin, Iran; 7 Department of Pharmacology, Qazvin University of Medical Sciences, Qazvin, Iran

**Keywords:** *Toxoplasma gondii*, *Ganoderma lucidum*, *In vitro*, Iran

## Abstract

**Objective:**

*Ganoderma* extracts have the potential to be used as anti-cancer, anti-inflammatory, immunomodulator, and antimicrobial agents, as evaluated in numerous studies. This study was aimed to determine the lethal and inhibitory effects of aqueous, hydroalcoholic, and alcoholic extracts of *Ganoderma lucidum* on *Toxoplasma gondii* RH strain tachyzoites, *in vitro*.

**Results:**

All three types of extracts showed toxoplasmacidal effects. The highest percentage of mortality was related to hydroalcoholic extract. The EC50 of *Ganoderma* extracts for tachyzoites were 76.32, 3.274, and 40.18 for aqueous, hydroalcoholic and alcoholic extracts, respectively. The selectivity index obtained for hydroalcoholic extract was 71.22, showing the highest activity compared to other extracts. According to our findings, the hydroalcoholic part was the most effective substance among the extracts. This basic study showed obvious anti-toxoplasma effect of *Ganoderma lucidum* extracts. These extracts can be used as candidates for further in-depth and comprehensive studies especially *In vivo* experiments to prevent toxoplasmosis.

## Introduction

*T. gondii* is an intracellular protozoan parasite which is the agent of toxoplasmosis [1]. The life cycle of the protozoan is divided into two sexual and asexual replication stages. The sexual replication stage occurs in nature, requiring a definitive host, to produce oocysts and then sporozoites. Once ingested by an intermediate host (vast number of domestic animals and humans), the parasites turn into tachyzoite form, which is responsible to induce toxoplasmosis [2]. Toxoplasmosis can occur by ingesting infectious oocysts, tissue cysts or tachyzoites, indicating that contaminated raw meat, milk, water, unwashed vegetables, and poor animal care could be considered as various risk factors of the disease [3]. Primary infection in pregnant women can cause congenital toxoplasmosis in fetus [4].

The parasite has a low host specificity therefore, large groups of human populations could be infected [5, 6]. The infection rate of the parasite poses medical, veterinary, food safety, and public health concerns, globally [7, 8]. The standard drugs commonly used for therapy are pyrimethamine, sulfadiazine or a combination of pyrimethamine and sulfadiazine [9, 10]. Unfortunately, these drugs are unable to kill the encysted form of the parasite and exhibit side effects such as neutropenia, thrombocytopenia, leucopenia, increase in serum levels of creatinine and liver enzymes, hematological abnormalities, and hypersensitivity reactions [8, 10, 11]. Herbal extracts have the potential to be used as alternative compounds which are considered as new generation of safe and effective drugs in toxoplasmosis, especially due to their antimicrobial activities. Nowadays, serious attention has been paid to research on medicinal fungi. *Ganoderma lucidum* (GL) is medical fungus with diverse properties including anti-cancer, anti-inflammatory, and immunomodulating activities [12, 13]. Some studies have examined the antimicrobial effect of this fungus [14–16]. The result of such studies showed that the aqueous extract of *G. lucidum* decreased the parasitemia levels of plasmodium in tissues [17]. Different fractions of the fungus were investigated against plasmodium and leishamania but no study was carried out regarding the anti- toxoplasma activity of this fungus [18–21]. The objective of the present study was to evaluate the efficacy of aqueous, hydroalcoholic, and alcoholic extracts of *G. lucidum* on *T. gondii* RH strain tachyzoites, *in vitro*.

## Materials and methods

### ***Ganoderma*** extracts and ***Toxoplasma*** parasite

***Ganoderma*extracts and*Toxoplasma*parasite**.

This study was carried out in 2021. The fungus *G. lucidum* was commercially purchased from a domestic distributor (Iran Ganoderma Company, Karaj Iran). The fungus was prepared in three forms including dried aqueous, hydroalcoholic, and alcoholic extracts by the National Genetics Center of Iran and confirmed by a botanist (Tehran, Iran). Briefly, the dried fungal specimens were cleaned and crushed and mixed with relevant solvent. Later, the samples were placed in a shaker incubator at a temperature of 40℃ for 24 h. The extract was filtered and the remaining solvent using was removed using a rotary evaporator (IKA, RV10, China). The dried aqueous, hydroalcoholic, and alcoholic extracts of Ganoderma fungus were dissolved in 1% dimethyl sulfoxide (DMSO) and RPMI1640 to prepare 10, 50, 100, 150, and 200 mg/ml concentrations [21]. Pyrimethamine (Sigma, USA) dissolved in methanol-acetone (50% v/v) and diluted with Roswell Park Memorial Institute (RPMI) 1640 medium was used as positive control. We used *T. gondii* RH strain tachyzoites. The tachyzoites were inoculated into the peritoneal cavity of BALB/c mice followed by harvesting the tachyzoites from peritoneal cavity 72 h after inoculation. Later, the fresh tachyzoites were washed 3 times with PBS phosphate buffered saline (PBS, pH 7.2). Finally, a suspension containing 10^6^ tachyzoites /ml was prepared and used in our experiments [22]. The tachyzoites possess 100% viability based on methylene-blue staining [23].

### Cell culture

The Vero cell line (ATCC Number: CCL-81) was purchased from the National Cell Bank of Iran (Pasteur Institute, Iran). The cell line was adapted to RPMI 1640 + 10% Fetal Bovine Serum (FBS, Gibco), supplemented with penicillin (100 IU/mL) and streptomycin (100 mg/mL). The growth condition of cells was optimized by incubating at 37 °C in 5% CO2 with culture passages every 72 h.

### Evaluating the activity of ***Ganoderma*** extracts on Vero cell line

To prepare Vero cells monolayer, the cells were washed with PBS, and then cultivated in sterile 96-well tissue culture plate 24 h before the experiment. Different concentrations of aqueous, hydroalcoholic, and alcoholic extracts (10, 50, 100, 150, and 200 mg/ml) were added to Vero cells monolayer to measure the toxicity of the extracts on the Vero cell alone following 24, 48, and 72 h incubation period. Positive control with pyrimethamine in 10 mg/ml (Sigma, USA) and negative control (Vero cell and RPMI1640) were used in the experiments. The cytotoxicity of various concentrations of fungal extracts was examined using MTT assay kits (Bio Idea Company, Tehran, Iran). All experiments were assayed in triplicate.

### Co-cultivation of ***T. gondii*** with vero cell line

Based on the results obtained for toxicity of extracts on Vero cells, two concentrations of 10 and 50 mg/ml of each extract were selected to evaluate their toxicity on tachyzoites survival. A suspension of 6 × 10^4^ cells/ml, counted in the exponential growth stage, was prepared and transferred to sterile 96-well tissue plate followed by adding a suspension of 3 × 10^5^ tachyzoites/ml to each well. Following an incubation period of 6 h at 37 °C and 5% carbon dioxide, the cultures were washed twice with RPMI1640 medium without fetal bovine serum (FBS) to remove non-adherent tachyzoites. Eighteen hours after incubation, the aqueous, hydroalcoholic, and alcoholic fraction were individually added to the wells at two concentrations of 10 and 50 µg/ml and incubated for 24, 48, and 72 h under the same conditions. Positive control with pyrimethamine in 10 mg/ml (Sigma, USA) and negative control were also used in the experiments. Anti-toxoplasma activity and cytotoxicity of those fungal extracts were surveyed using MTT assay kits (Bio Idea Company, Tehran, Iran). Optical densities (ODs) were read using an enzyme-linked immunosorbent assay (ELISA) microplate reader (Epoch, USA) in the wavelength of 570 nm. All experiments were examined in triplicate. The effective concentration of EC50 was determined by using Prism software (version 5.04) and selectivity Index determined for aqueous, hydroalcoholic, and alcoholic extracts of *Ganoderma*. Statistical analysis on data was carried out using one way ANOVA for cell viability of tachyzoites and independent T test for mortality of tachyzoite. The significance level was P < 0.05.

## Results

**Fig. 1:**
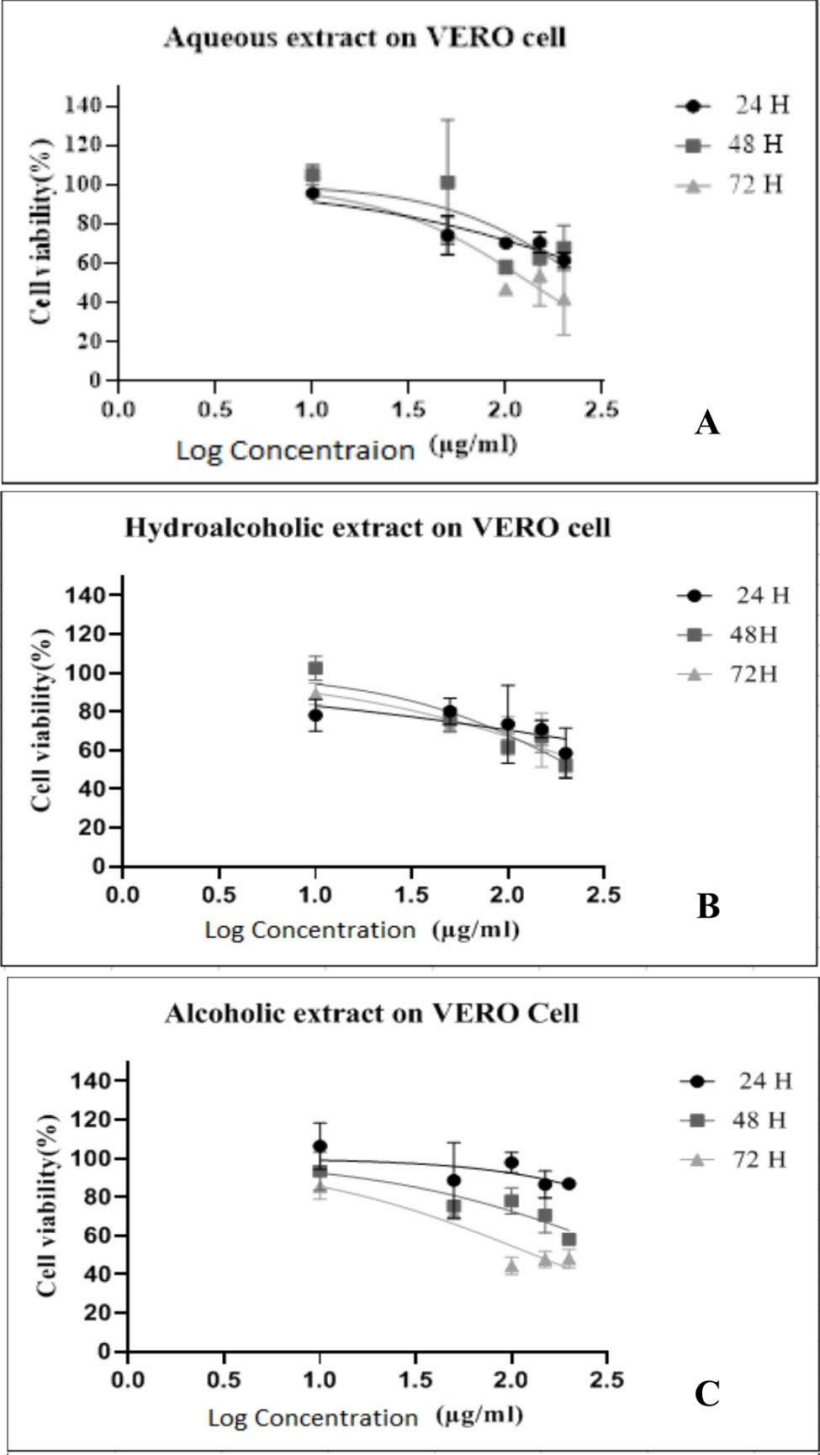
Cell viability percentage of Vero cells at different concentrations of aqueous (A), hydroalcoholic (B) and alcoholic (C) extracts of *Ganoderma lucidum* according to different contact times

**Fig. 2:**
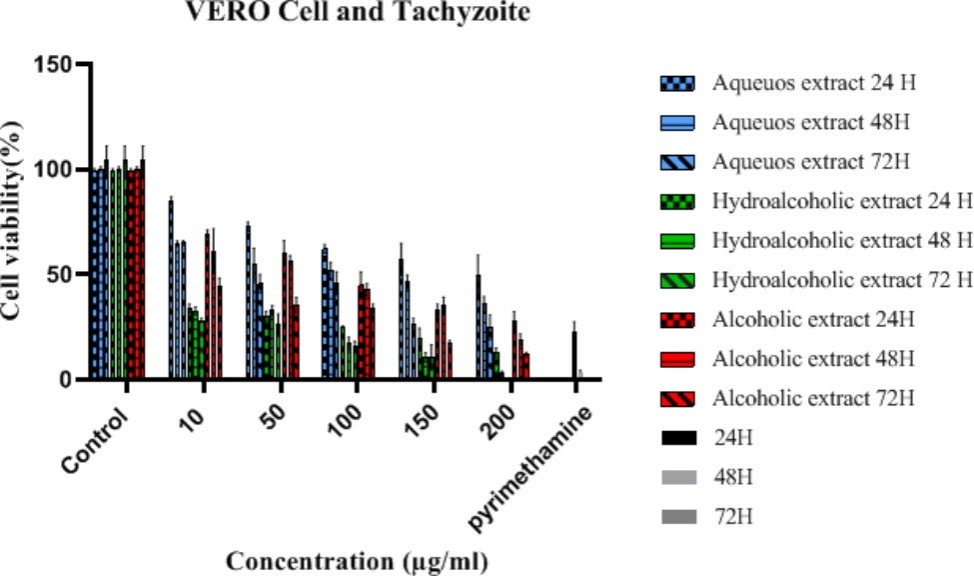
Cell viability percentage of Vero + tachyzoites cells at different concentrations of aqueous, hydroalcoholic, and alcoholic extracts of *Ganoderma lucidum* according to different contact times

The cell viability following treatment of Vero cells with three extracts is shown in Fig. 1. The percentage of cell viability decreased with an increase in concentration and proximity time. The cell viability following the addition of three extracts on Vero cells + tachyzoites is shown in Fig. 2. All three extracts of the *Ganoderma* showed toxoplasmacidal effects. The results of mortality rates (%) following the addition of aqueous, hydroalcoholic, and alcoholic extracts of *Ganoderma* on tachyzoites are shown in Table 1. The highest percentage of mortality rate was related to hydroalcoholic extract. The difference in the mortality rates of hydroalcoholic extract to other two fractions was significant (P < 0.001) (Table 1). The EC50 of *Ganoderma* extracts on Vero cell and tachyzoites is clarified in Table 2. The SI values for aqueous, hydroalcoholic, and alcoholic extracts of *G. lucidum* were 2.96, 71.2, and 10.93, respectively. The hydroalcoholic extract was found to have the highest effect on tachyzoites.

**Table 1:** Mortality (%) of *T. gondii* RH strain tachyzoites after 48 h treatment with aqueous, hydroalcoholic, and alcoholic extracts of *Ganoderma lucidum*

Concentration (µg/ml)	Hydroalcoholic extracts	Aqueous extract	*P.value*	Alcoholic extracts	*P.value*
10	68 ± 1.82	36 ± 1.27	< 0.001	39 ± 3.53	< 0.001
50	68 ± 2.40	46 ± 7.53	< 0.009	44 ± 2.46	< 0.001
100	83 ± 2.85	49 ± 4.01	< 0.001	58 ± 2.85	< 0.001
150	90 ± 2.28	54 ± 3.36	< 0.001	65 ± 3.83	< 0.001
200	97 ± 1.14	65 ± 3.56	< 0.001	81 ± 2.63	< 0.001

**Table 2:** In vitro anti-toxoplasma activity and selectivity of *Ganoderma lucidum* extracts

*Ganoderma Lucidum* extract	EC50 (µg/ml)		Selectivity index
	VERO	VERO + *T. gondii*	
**Aqueous**	226.3	76.32	2.96
**Hydroalcoholic**	233.2	3.274	71.22
**Alcoholic**	439.5	40.18	10.93

**Fig. 3:**
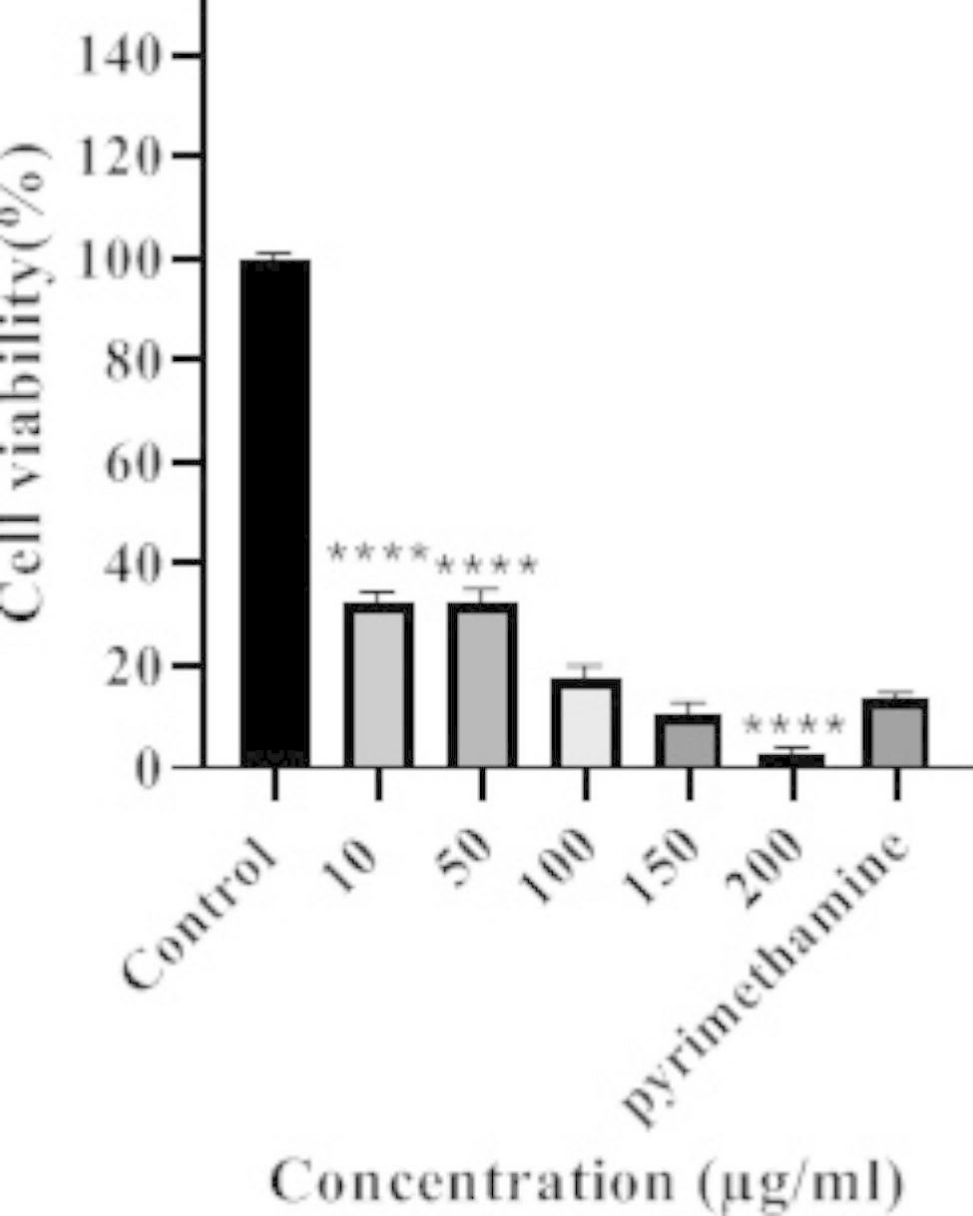
Inhibitory effect of *G. lucidum* extract on *T. gondii* tachyzoites in cell cultures

The cell viability of *T. gondii* for hydroalcoholic extract was significant at 10, 50, and 200 µg/ml concentrations, compared to pyrimethamine. The two concentrations of 10 and 50 µg/ml of hydroalcoholic extract caused significant increase compared to pyrimethamine, while at 200 µg/ml concentration, there was a significant decrease when compared to pyrimethamine (Fig. 3).

## Discussion

All three extracts of *Ganoderma* showed toxoplasmacidal effects. This is the first study regarding the anti-toxoplasma activities of the extract although several studies have shown anti-parasitic properties of *Ganoderma* extracts on *plasmodium*, *leishmania*, and *trypanosome* [18, 24–26]. It is demonstrated that the aqueous fraction of *G. lucidum* has the ability to diminish the serum and liver lipoprotein cholesterol contents, a finding correspond to a reduction in the degree of parasitemia and tissue infection (Oluba and colleagues, 2012). Moreover, It is also reported that the ethanolic extract of this fungus has antimalarial properties, leading to better improvement of accompanying liver damage induced by plasmodium infection [17, 20]. Crude chloroform extract of *G. lucidum* could reduce parasitemia and improve the attendant consequences of *Plasmodium berghei* infection in mice [26]. The result of another study declares that aqueous extract of *G. lucidum* possesses potent antioxidant activity which protects hemoglobin against *Plasmodium*-induced oxidative damage [27]. Also, a remarkable antiplasmodial activity of ethyl acetate extract of *G. lucidum* at 4.9 µg/ml concentration with 79% inhibition is reported [24]. Otokpa Ede and colleagues indicated that the aqueous extract of *G. lucidum* is a potential source of trypanocidal compounds [25]. So far, the alcoholic, chloroform, aqueous, and polysaccharide extracts of *G. lucidum* have been used on a wide range of microorganisms and the results show an inhibitory effect of the extracts on the growth of microbial agents [28, 29].

Polysaccharides, steroids, terpenoids and triterpenes have been identified in the *Ganoderma* component [30–33]. The skeletons of triterpenoids and farnesyl quinone type of *Ganoderma* has antimicrobial and antiparasitic activity [34]. The results of another study indicate that the antiplasmodial activity of aqueous extract may be attributed to the presence of terpenes, sterols, and flavonoids in the extract [17].

The results of this study showed that the highest mortality was related to hydroalcoholic extract. According to the results obtained for selectivity index, a value of 71.22 was calculated for hydroalcoholic extract, indicating a higher effect compared to other two extracts. The findings of a study performed by HPLC method indicate the presence of high amount of phenolic and flavonoid compounds in the hydroalcoholic extract, emphasizing on antioxidant property for this fungus. On the other hand, this extract can reduce lipopolysaccharides-induced cytokines including TNF-α, IFN-γ, IL-1β, and suppressed NO, which can play an effective role in relieving inflammatory diseases [35]. In some studies, the radioprotective activity of hydroalcoholic extract in mammalian organism, and hypoglycemic and hypolipidemic properties have also been reported for this extract [36, 37]. The only study of hydroalcoholic extract of *Ganoderma* performed on in parasites revealed that high concentrations (150 and 200 mg/ml) of the extract could significantly inhibit and reduce the growth of *leishmania major* [18]. There are limited number of studies concerning the effect of hydroalcoholic extract of several plants on *T. gondii*, such as Brassicaceae species and *Terminalia chebula*, evaluated for anti-toxoplasma activities [38, 39]. One of the most important challenges in toxoplasmosis research is to find an effective drug with fewer side effects, compared to common synthetic drugs, for the treatment of this disease. This still remains an ongoing challenge as despite extensive research, this goal has not been achieved so far. The findings of the present study showed that the hydroalcoholic extract of *G. lucidum* may be used as a candidate for further studies in preventing the occurrence of toxoplasmosis.

## Conclusion

The results of this study showed that the anti-toxoplasma activities of *G. lucidum* extracts. The highest percentage of mortality and the result obtained for selectivity index indicate that the hydroalcoholic extract is more effective than other two extracts. Therefore, these extracts could to be good candidates for future anti-toxoplasma studies.

## Limitation

Due to the unfavorable conditions of the animal house, we could not perform this study *in vivo* condition.

## Data Availability

All data generated or analysed during this study are included in this published article.

## Abbreviations

*T. gondii*: *Toxoplasma gondii*
MTT: (3-(4, 5-dimethylthiazol-2-yl)-2, 5-diphenyltetrazolium bromide)
EC50: Effective concentration
RPMI1640: Roswell Park Memorial Institute Medium
